# Role of endoscopic ultrasound elastography in differential diagnosis of pancreatic solid masses

**DOI:** 10.5339/qmj.2021.40

**Published:** 2021-09-07

**Authors:** Bashar Almasri, Ayman Ali

**Affiliations:** Damascus University, Damascus, Syria E-mail: dr.bashar.m@gmail.com

**Keywords:** EUS elastography, pancreas solid masses, pancreas tumors, adenocarcinoma of the pancreas, qualitative and quantitative elastography

## Abstract

Introduction: Endoscopic ultrasound (EUS) elastography is another technique that measures the stiffness of tissue and adds more diagnostic value to EUS.

Objective: This study aimed to assess the ability of qualitative and quantitative EUS elastography in differentiating malignant from benign solid pancreatic masses.

Methods: This 2-year cross-sectional study enrolled 80 patients with solid pancreatic masses in the department of endoscopy in Alassad University Hospital who underwent conventional and elastography-assisted EUS and then followed for pathology through EUS-guided or CT-guided biopsy or surgery.

Results: Qualitative elastography using a 5-point scoring system was able to recognize malignant pathology (obtained by EUS-guided biopsy, CT-guided biopsy, or surgery) with a sensitivity, specificity, and accuracy rates of 100%, 28.6%, and 81.3%, respectively. A quantitative method using hue histogram had a sensitivity of 71.2%–86.4% and specificity of 71.4%–81% with the best accuracy for histogram mean ratio (area under the curve, 0.867).

Conclusion: EUS elastography is a simple and good alternative method in differentiating malignant from benign pancreatic solid masses.

## Introduction

Majority of pancreatic tumors arise from epithelial cells, with adenocarcinoma being the most prevalent (90%).^1^ Other epithelial tumors include cystic tumors and intraductal papillary mucinous neoplasms. Non-epithelial tumors include neuroendocrine tumors (NETs), mesenchymal tumors, and metastatic tumors.^2^ Some non-neoplastic lesions can manifest as masses such as inflammatory pseudotumors.^3^ Pancreatic tumors are diagnosed through imaging techniques such as contrast-enhanced multi-slice computed tomography (CT), magnetic resonance imaging, or endoscopic ultrasonography (EUS). Pancreatic mass types are difficult to distinguish from each other using imaging techniques only. The gold standard for the diagnosis is pathology, which can be obtained by CT-guided or EUS-guided biopsy, but both have limitations and risks.^4^

Elastography is an additional option embedded in new EUS devices. Elastography is designed to measure the stiffness of tissues, depending on the principle of shape deformation under straining caused by the pressing EUS probe. This deformation can be monitored and recorded on B-mode EUS images as colors, ranging from red (softest) through green (soft) to blue (hard). The picture should stay stable for 5 s at least to be reliable.^5^

Elastography can be used to assess pancreatic masses in two ways. First, qualitative elastography method includes using a 5-point scoring system according to the mass color pattern, with the following scale scores: 1 point, homogeneous green and compatible with normal tissue; 2 points, heterogeneous (green, yellow, and red) and compatible with fibrosis; 3 points, mostly blue with minimal heterogeneity and compatible with early adenocarcinoma; 4 points, blue with a central green hypoechoic region and compatible with NET or metastasis; 5 points, blue with heterogeneity due to necrosis and compatible with late adenocarcinoma.^6^ Second, quantitative elastography method include the use of strain ratio (ratio between the stiffness of the lesion and an adjacent red region) and hue histogram (graphical color distribution on a two-axis, the x-axis represents stiffness between 0 and 255, and the y-axis represents the number of pixels of each elasticity level in the region of interest).^7^ This study was designed to assess the ability of EUS elastography in differentiating benign from malignant pancreatic solid masses.

## Methods

A prospective cross-sectional study was undertaken in the endoscopy department in Alassad University Hospital in Damascus, Syria, during the period from April 2018 to March 2020, on patients with pancreatic solid masses that were assessed with EUS. The inclusion criteria were as follows: pancreatic masses diagnosed by CT after onset of obstructive jaundice or abdominal pain. The exclusion criteria were as follows: inability to take biopsy or follow the pathology of pancreatic mass and the presence of a contraindication of the EUS procedure.

The procedure was clearly explained to the patients, and informed consent was signed before the procedure. The procedure was performed under medical sedation without tracheal intubation. All patients were in a good medical condition before, during, and after the procedure, without complications, except for mild abdominal pain in 10 patients after taking fine-needle aspiration (EUS-FNA) which was alleviated by intravenous administration of acetaminophen.

The study protocol was reviewed by the National Committee on the Ethics of Scientific Knowledge and Technology in Syria, and approval was obtained (no. 19-003-1).

**Figure 1.**
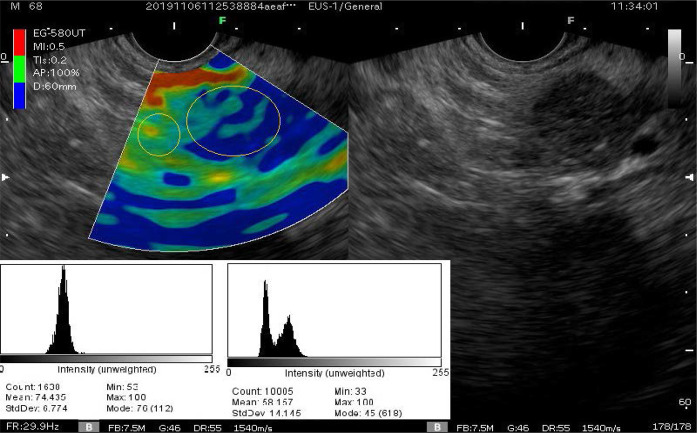
Hue histogram generated with ImageJ application

The study was performed using an endoscopic ultrasound system from Fujinon™ (Tokyo, Japan). The procedure was performed by two endoscopists; one of them is an EUS expert (who has performed >1000 diagnostic and therapeutic EUS procedures). The radial endoscope was frequently used, except for cases that acquired EUS-guided FNA. The mass was assessed first with conventional B-mode to evaluate the characteristics of the mass and adjacent tissues, and color elastography was then applied on a clear section showing the mass and a sufficient region of the normal pancreatic tissue. The color view should be stable for at least 5 s to guarantee a valid and reliable image. When allocating the area of interest, avoidance of large vessels, cystic lesions, and clear calcifications was attempted to decrease possible color confusion. The qualitative elastography score was recorded according to the 5-point scoring system under the agreement of the two endoscopists. The 5-point scoring system was not validated before use, since it was studied well in previous studies.^5^ EUS elastography images were processed using ImageJ v 1.52a application (Image J software, NIH, Bethesda, MD, USA)^8^ to generate a hue histogram for the mass region and a region of normal tissue with the same distance from the probe (Figure 1), avoiding ducts, vascular, or cystic components that may cause confusion during elastography. Pathological findings were followed up after obtaining tissue samples from EUS-FNA, CT-guided biopsies, or surgery.

### Statistical analysis

Statistical analysis was performed using SPSS v.20 (IBM, Armonk, NY, USA) to calculate means, sensitivity, specificity, negative predictive value (NPV), and positive predictive value (PPV); compare means using t-test for independent samples; create receiver operating characteristic (ROC) curves and calculate the area under the curve (AUC), and identify cutoff points with the best sensitivity and specificity. A P-value of less than 0.05 was considered evidence for significant results.

## Results

A total of 105 patients with a pancreatic solid mass on EUS were enrolled in this study. However, 25 patients were dropped because of the inability to take a EUS-guided, CT-guided, or surgical samples for pathology. Therefore, 80 patients were included in the final study sample. Samples for pathology were collected without adverse events, with 23 EUS-guided biopsies, 41 CT-guided biopsies, and 16 surgically obtained samples.

Most patients were male (53.75%) and came from rural areas (57.5%). The mean age of the patients was 58.6 years. About one-third of the patients had diabetes, and the most common risk factor for pancreas cancer was smoking (83.75%). General data from the study are illustrated in Table 1.

**Figure 2.**
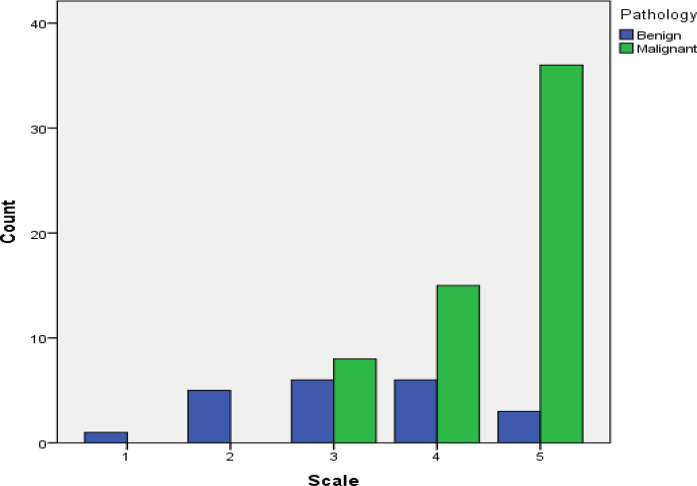
Distribution of color scale according to pathological findings

When EUS elastography was applied, majority of the masses showed a hard pattern (using the 5-point scoring system) with 41 masses having a score of 5 points, 21 with 4 points, 15 with 3 points, 2 with 2 points, and one with 1 point. Results of the correlation analysis of the color pattern with pathological findings are shown in Table 2 and Figure 2.

Assuming that 1 and 2 points on the color scale refer to benign findings and 3, 4, and 5 points indicate malignant ones,^10^ the diagnostic value of the color scale in solid pancreatic masses is shown in Table 3.

Hue histograms for the mass area and an adjacent normal pancreatic area were generated, and calculated quantitative values are represented as mean (mean of all values represented in the histogram), mode (most frequent value in the histogram), mean ratio (ratio of the mean value of the mass area to the mean value of the normal area), and mode ratio (ratio of the mode value of the mass area to the mode value of the normal area). Table 4 and Figure 2 illustrate the quantitative values according to the pathological findings.

With regard to the ability of quantifying the expectation for a mass to be malignant, independent sample t-test was used to compare calculated values between malignant and benign lesions in Table 5.

**Figure 3.**
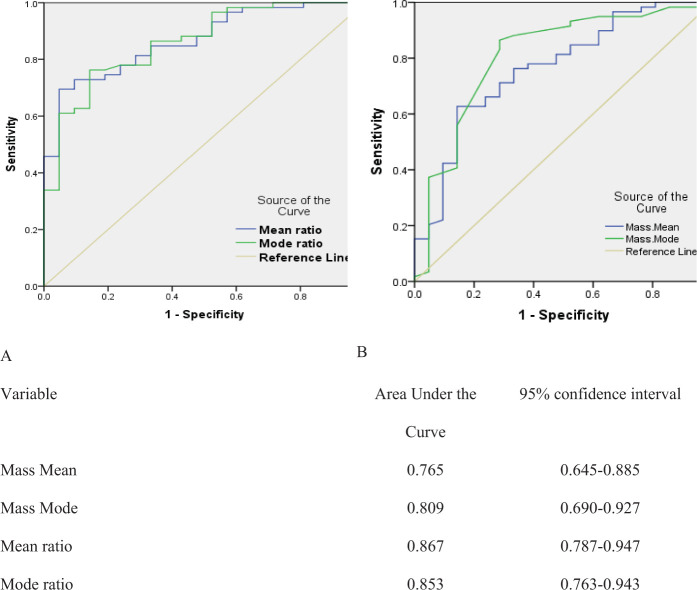
ROC curves for elastography quantitative variables in expecting malignant lesions. A: Mean and mode ratio, B: Mean and mode of mass

Using the ROC curve, the accuracy of these values was calculated as illustrated in Figure 3.

From the ROC curve, the cutoff points that were compatible with the best sensitivity and specificity were extracted as shown in Table 6.

## Discussion

Pancreatic masses frequently represent diagnostic challenges owing to the difficulty in exposing the lesion and obtaining biopsies.^4^ EUS is a helpful diagnostic investigation that exposes the pancreatic lesions clearly because of the proximity between the probe and the pancreatic tissue. Moreover, it offers a good way to take pathological samples through EUS-FNA and biopsy. The possible complications from obtaining pancreatic biopsies include pain, pancreatitis, hemorrhage, and tumor seeding.^9^ Elastography is an additional option that can increase diagnostic accuracy, so it can spare the need for biopsy and thereby avoid adverse events.

There were more male than female patients (53.75% vs. 46.25%), and more patients came from rural areas than from urban areas (57.5% vs. 42.5%). The mean age of the patients was approximately 58 years, which agrees with the usual late onset of pancreatic masses.^4^ The most common accompanying medical problem was diabetes mellitus (nearly one-third of the patients), which is a usual risk factor in patients with pancreatic cancer.^4^ Most of the patients were smokers (approximately 84%), reflecting the wide prevalence of smoking and the role of smoking as a risk factor for pancreatic disease.^4^

As regards the distribution of pancreatic lesions, about three-quarters of patients had malignant lesions, with adenocarcinoma being the most prevalent. The most commonly observed benign lesion demonstrated chronic inflammation, which were showed similar distribution between malignant and benign lesions in other studies.^5,6^

The study can be divided into two main parts: testing qualitative (color pattern) elastography in differentiating benign and malignant masses and studying quantitative elastography (hue histogram) for the same purpose.

As regards qualitative elastography, most of the lesions (92.5%) demonstrated hard patterns (3, 4, and 5) even with benign lesions. This was responsible for the low specificity owing to a high false-positive ratio. Malignant lesions never demonstrated a soft color pattern (0 cases), leading to a high NPV of color pattern. A high NPV means that qualitative EUS elastography is a good test for excluding malignancy.

After generating a hue histogram, values of qualitative variables were lower in malignant lesions than in benign lesions, demonstrating a significant difference. In the comparison of the lesions with normal tissue using semi-quantitative values, both mean and mode ratios are significantly larger in malignant lesions.

The ROC curves showed that the mean ratio is the most accurate method to differentiate malignant from benign pancreatic lesions, with the best AUC. Using the mean ratio with 1.4 as a cutoff point gave the best sensitivity and specificity. These new variables (mean ratio and mode ratio), which were barely investigated previously, can add more value to accurately differentiate malignant from non-malignant lesions.

The sensitivity of the qualitative method was high in all other studies, similar to our finding (100%). Sensitivity ranged from 92% to 100%. Our study showed a 28.6% specificity, which is lower than that in other studies, with specificity between 33% and 86%.^5,6,10–13^ Some studies have used the 5-point scoring system (similar to what we used),^5,10,12,13^ while others have used the 3-point scoring system.^6,11^

Moreover, the qualitative histogram method showed good sensitivity of 71.2%–86.4%, but lower than those in other studies (85%–100%),^7,14–18^ and good specificity (71.4%–81%) when compared with those of other studies (45%–92%).^7,14–18^ More variables associated with histogram were generated and tested using mass mean, mass mode, mean ratio, and mode ratio, while other studies have focused mainly on the mean value.

## Limitations

This was a single-center study with only two endoscopists assessing the cases; thus, more multicenter studies employing more endoscopists are required to obtain more accurate results, especially about interobserver variation in assessing color pattern.

Some difficulties were encountered when applying color elastography, such as patient's respiration effort during imaging and the inability to reproduce the same color pattern. Applying the elastography technique increased the imaging time by about 3 minute on average. In some cases, elastography requires longer time, so the endoscopist was forced to cancel it in patients with high risk for anesthetics used, where a quick procedure is needed.

## Conclusion

EUS elastography (both qualitative and quantitative) is a new, simple, relatively safe, and minimally invasive tool with good results in assessing hardness and malignant tendency of pancreatic masses. It provides an additional option in differentiating malignant from benign pancreatic solid masses, saving some patients from risks of obtaining unnecessary biopsies.

### Conflict of interest

The authors declare that there is no conflict of interest regarding the publication of this article.

## Figures and Tables

**Table 1:** Demographic and medical characteristics of sample patients and pathological findings of pancreatic masses

Gender (male/female)		43 (53.75%)/37 (46.25%)	

Mean age ± standard deviation		58.6 ± 12.6 years	

Area of residence (urban/rural)		34 (42.5%)/46 (57.5%)	

Marital status (married/single or widow)		63 (78.75%)/17 (21.25%)	

Other medical problems (diabetes/hypertension/cardiovascular diseases/past malignancies)		25 (31.25%)/18 (22.5%)/10 (12.5%)/2 (2.5%)	

Pancreatic cancer risk factors (smoking/alcoholism/chronic pancreatitis)		67 (83.75%)/3 (3.75%)/5 (6.25%)	

Pathology			

Malignant lesions	Adenocarcinoma	56 (70%)	59 (73.75%)

	Gastrinoma	1 (1.25%)	

	NET	1 (1.25%)	

	IPMN	1 (1.25%)	

Benign lesions	Chronic inflammation	18 (22.5%)	21 (26.25%)

	Angiolipoma	1 (1.25%)	

	Insulinoma	2 (2.5%)	


**Table 2:** Distribution of the color scale according to the pathological findings

		Pathology							

		Malignant				Benign			Total

		Carcinoma	Gastrinoma	IPMN	NET	Chronic inflammation	Insulinoma	Angeolipoma	

Scale	1	0	0	0	0	0	1	0	1

	2	0	0	0	0	5	0	0	5

	3	7	0	0	0	5	0	1	14

	4	14	0	1	1	5	1	0	21

	5	35	1	0	0	3	0	0	39

Total		56	1	1	1	18	2	1	80


**Table 3:** Diagnostic values of the 5-scale color score of qualitative elastography in differentiating benign from malignant solid pancreatic masses.

Sensitivity	Specificity	NPV	PPV	Accuracy

100%	28.6%	100%	79.7%	81.3%


**Table 4:** Distribution of elastography quantitative values according to the pathological findings

Pathology	Mass mean	Mass mode	Mean ratio	Mode ratio

	Mean ± std. dev			

Adenocarcinoma	48.6 ± 6.3	43.6 ± 6.4	1.58 ± 0.2	1.71 ± 0.3

Chronic inflammation	54.9 ± 8.1	49.9 ± 8.2	1.26 ± 0.2	1.31 ± 0.2

Insulinoma	64.4 ± 12.6	64 ± 16.9	1.23 ± 0.3	1.28 ± 0.5

Gastrinoma	59.5	39	1.16	1.62

NET	38.9	38	1.78	1.89

IPMN	46.3	41	1.47	1.61

Angiolipoma	61.5	49	1.18	1.37

All cases	50.6 ± 7.8	45.4 ± 8	1.49 ± 0.3	1.6 ± 0.3


**Table 5:** Comparison of mean values of elastography between malignant and benign lesions

Variables	Mean ± std. dev		P-value

	Malignant lesion	Benign lesion	

Mass mean	48.6 ± 6.4	56.2 ± 8.6	< 0.01

Mode mean	43.3 ± 6.3	51.2 ± 9.5	< 0.01

Mean ratio	1.57 ± 0.2	1.26 ± 0.2	< 0.01

Mode ratio	1.71 ± 0.3	1.31 ± 0.2	< 0.01


**Table 6:** Diagnostic values and cutoff points for elastography quantitative variables

Variable	Cutoff point	Sensitivity	Specificity

Mass Mean	51.69	71.2%	71.4%

Mass Mode	47.5	86.4%	71.4%

Mean Ratio	1.40	78%	77.2%

Mode Ratio	1.48	76.3%	81%

